# A Tri-Marker Proliferation Index Predicts Biochemical Recurrence after Surgery for Prostate Cancer

**DOI:** 10.1371/journal.pone.0020293

**Published:** 2011-05-23

**Authors:** Sameer Malhotra, Jacques Lapointe, Keyan Salari, John P. Higgins, Michelle Ferrari, Kelli Montgomery, Matt van de Rijn, James D. Brooks, Jonathan R. Pollack

**Affiliations:** 1 Department of Urology, Stanford University, Stanford, California, United States of America; 2 Department of Surgery, Urology Division, McGill University, Montreal, Quebec, Canada; 3 Department of Pathology, Stanford University, Stanford, California, United States of America; 4 Department of Genetics, Stanford University, Stanford, California, United States of America; Baylor College of Medicine, United States of America

## Abstract

Prostate cancer exhibits tremendous variability in clinical behavior, ranging from indolent to lethal disease. Better prognostic markers are needed to stratify patients for appropriately aggressive therapy. By expression profiling, we can identify a proliferation signature variably expressed in prostate cancers. Here, we asked whether one or more tissue biomarkers might capture that information, and provide prognostic utility. We assayed three proliferation signature genes: *MKI67* (Ki-67; also a classic proliferation biomarker), *TOP2A* (DNA topoisomerase II, alpha), and *E2F1* (E2F transcription factor 1). Immunohistochemical staining was evaluable on 139 radical prostatectomy cases (in tissue microarray format), with a median clinical follow-up of eight years. Each of the three proliferation markers was by itself prognostic. Notably, combining the three markers together as a “proliferation index” (0 or 1, *vs.* 2 or 3 positive markers) provided superior prognostic performance (hazard ratio = 2.6 (95% CI: 1.4–4.9); *P* = 0.001). In a multivariate analysis that included preoperative serum prostate specific antigen (PSA) levels, Gleason grade and pathologic tumor stage, the composite proliferation index remained a significant predictor (*P* = 0.005). Analysis of receiver-operating characteristic (ROC) curves confirmed the improved prognostication afforded by incorporating the proliferation index (compared to the clinicopathologic data alone). Our findings highlight the potential value of a multi-gene signature-based diagnostic, and define a tri-marker proliferation index with possible utility for improved prognostication and treatment stratification in prostate cancer.

## Introduction

Prostate cancer is a leading cause of cancer death in the United States [1]. Despite that, prostate cancer exhibits considerable variability in clinical behavior. Many (if not most) prostate cancers are clinically indolent, while others are clinically aggressive, becoming metastatic and lethal. For localized prostate cancer, treatment options range from active surveillance (“watchful waiting”) to decisive surgical excision (radical prostatectomy) or radiation therapy [2], [3]. Docetaxel chemotherapy is also being evaluated in the adjuvant and neoadjuvant setting [4]. Increasingly, there is a need for prognostic biomarkers to accurately stratify patients for appropriate risk-adapted therapy.

Current prognostic factors include Gleason grade, tumor stage, and preoperative serum PSA [5]–[8], and nomograms that combine such data [9], [10]. However, much uncertainty remains, and as such many men opt for unnecessarily aggressive therapy. Other measures, e.g. tumor histology, DNA ploidy, proliferation markers, and selected cancer genes, are also under evaluation [11]–[15]. Of these, markers of tumor cell proliferation, e.g. S-phase fraction or Ki-67 immunostaining, are already in clinical use for other cancer types, including for example breast cancer [16], gastroentero-pancreatic neuroendocrine tumors [17], and mantle cell lymphoma [18].

In prior studies, we used DNA microarrays to profile gene expression in prostate cancer [19]. Unsupervised clustering of expression profiles revealed distinct tumor clusters (defining molecular subtypes), as well as distinct clusters (or “features”) of co-expressed genes, identifying the underlying biological pathways and processes. Among these, one prominent gene-expression feature ostensibly reflected tumor cell proliferation levels, and its expression varied substantially across prostate cancers. Here, we set out to characterize proliferation-signature genes as tissue biomarkers, and to evaluate their prognostic utility.

## Methods

### Molecular signatures

To evaluate a cell proliferation signature in prostate cancer, we analyzed previously published [19] cDNA microarray gene-expression profiles from 71 prostate cancer and 41 normal prostate (uninvolved tissue from the opposite lobe) specimens. The microarray data are MIAME compliant, and the raw data are accessible at GEO (accession GSE3933). Hierarchical clustering [20] was applied to the 7,957 genes (cDNAs) that were both well measured (signal/background >1.5 in at least 50% of specimens) and variably expressed (≥2-fold expression change from the median in at least 2 specimens). A gene cluster that included many genes with known roles in cell proliferation (a putative “proliferation signature” cluster) was further evaluated. Overlaps between the putative proliferation-signature cluster (containing 94 unique genes) and 639 canonical pathway (BioCarta and KEGG) gene sets were evaluated using the Molecular Signatures Database (MSigDB) [21] “compute overlap” function, based on the hypergeometric distribution. Ki-67, TOP2A and E2F1 were selected from among the proliferation signature genes based on the availability of antibodies suitable for immunohistochemistry on formalin-fixed, paraffin-embedded tissue.

### Tissue microarray cohort

A tissue microarray included 139 fully evaluable formalin-fixed, paraffin-embedded primary prostate tumors selected from diagnostic radical prostatectomy cases performed at Stanford University Hospital between 1986 and 1996, with Institutional Review Board approval and written patient consent (protocol ID 11612) [19]. Each case was represented by duplicate 0.6 mm tumor cores. Clinicopathological characteristics of the cases are summarized in Table 1. Patients were treated by radical prostatectomy alone, and clinical follow-up every 6 months included PSA testing and physical examination; the median clinical follow-up was 8 years.

**Table 1:** Clinicopathological characteristics of tissue microarray cases.

Clinicopathologic feature	Number of cases (%)
Preoperative PSA (ng/ml)	
0–10	83 (60%)
10–20	44 (32%)
≥20	12 (9%)
Gleason grade^a^	
3+3	20 (14%)
3+4	95 (68%)
4+3	22 (16%)
≥4+4	2 (1%)
Pathologic stage	
T2	13 (9%)
T2b	65 (47%)
T3a	42 (30%)
T3b	11 (8%)
T4	8 (6%)

a Determined from pathology report (not tissue microarray core).

### Immunohistochemistry

Serial sections of 4 µm were cut from the tissue microarray block, de-paraffinized in Citrisolv (Fisher Scientific, Pittsburgh, PA), and hydrated in a graded series of alcohol solutions. Heat-induced antigen retrieval was performed by microwave pretreatment in citrate (1 mM, pH 6.0) for 15 minutes before staining. Endogenous peroxidase was blocked by preincubation with 1% hydrogen peroxide in phosphate-buffered saline. Ki-67 mouse monoclonal antibody (MIB-1; Dako, North America, Carpinteria, CA) was used at 1∶100 dilution, TOP2A mouse monoclonal antibody (SWT3D1; Research Diagnostics, Flanders, NJ) at 1∶10 dilution, and E2F1 mouse monoclonal antibody (18E10; GeneTex, Irvine, CA) at 1∶200 dilution, each for 30 min. Chromogenic detection was carried out using a peroxidase-conjugated secondary antibody and DAB reagents provided with the Envision detection kit (Dako). Immunostains were scored by a pathologist (J.P.H) blinded to clinical data, and the criteria for positivity set to provide a dynamic range. For Ki-67 and TOP2A, strong staining in ≥5% tumor nuclei was scored as positive. For E2F1, which stained a higher proportion of nuclei, strong staining in ≥50% tumor nuclei was scored as positive. If either duplicate core scored positive, the case was considered positive.

### Statistical analysis

Correlations between proliferation biomarkers were assessed by Pearson correlation. Associations between proliferation biomarkers and clinicopathologic variables were evaluated by Fisher's exact test. Kaplan-Meier plots and the log-rank test were used to test the equality of the survivor functions across prostate cancer groups. Multivariate Cox proportional hazards regression analysis, with ties handled by the Efron appoximation, was used to identify the significant predictors of PSA recurrence. Receiver-operating characteristic (ROC) curves were evaluated for the significant predictors of PSA recurrence. All analyses were performed using the R statistical software package and WinStat software (R. Fitch Software, Chicago, IL).

## Results

**Figure 1:**
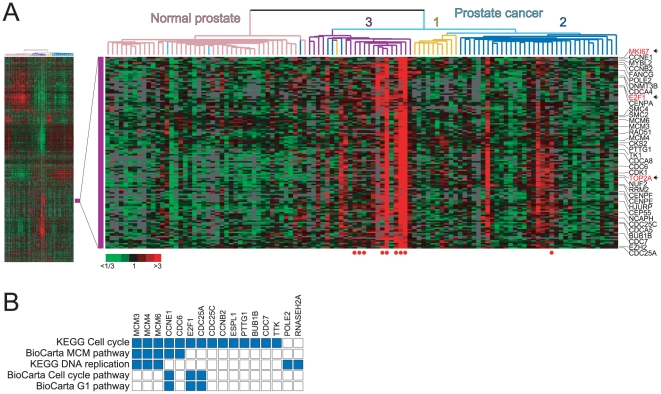
A proliferation signature cluster in prostate cancer. (**A**) Unsupervised cluster analysis of prostate cancers (data set from ref. [19]) reveals molecular subtypes of prostate cancer (1, 2 and 3, labeled), and gene-expression features reflecting underlying biological processes. *Left*, thumbnail heatmap of the cluster analysis. *Right*, enlarged view of the “proliferation cluster”, with selected genes shown (*MKI67*, *TOP2A* and *E2F1* in red text, marked by arrow). Red and green expression levels reflect high and low values, respectively (see key). Red filled circles (*below*) identify lymph node metastases. (**B**) Overlap matrix of proliferation cluster genes (N = 94) with canonical pathway (CP) gene sets identifies top gene set matches (all significant, *P*<0.001) all relating to cell-cycle/proliferation. Solid blue fill indicates overlapping membership between proliferation cluster and queried gene sets.

In our prior studies, expression profiling of prostate cancers revealed distinct molecular subgroups, as well as gene-expression features reflecting underlying biological processes [19]. In a re-analysis of those data, one prominent gene-expression feature (Fig. 1A) included genes regulating the cell-cycle (e.g. *CCNE1*, *CDK1*, *CDC25A*), and involved in DNA replication (e.g. *POLE2*, *MCM4*, *TK1*) and chromosome dynamics (e.g. *SMC4*, *CENPE*), ostensibly reflecting tumor cell proliferation levels. This feature was variably expressed across prostate cancers, and particularly prominent among lymph node metastases, and a subset of primary tumors within molecular subtypes 2 and 3, subgroups previously found to be more clinically-aggressive [19]. Evaluating overlaps between this cluster of genes and canonical pathway gene sets within the Molecular Signature Database [21] confirmed a connection to cell proliferation; the top five significantly (*P*<0.01) overlapping gene sets all related to cell-cycle/proliferation pathways (Fig. 1B).

High proliferation rates in cancers are typically associated with worse clinical outcome. We therefore sought to evaluate the prognostic value of proliferation signature genes, individually and in combination, in prostate cancer. Based on the availability of antibodies suitable for immunohistochemistry on formalin-fixed, paraffin-embedded tissue, we chose to focus on three proliferation signature genes, *MKI67* (encoding the classic cell proliferation marker, Ki-67) [22], *TOP2A* (DNA topoisomerase II, alpha), and *E2F1* (E2F transcription factor 1) (Fig. 1A).

**Figure 2:**
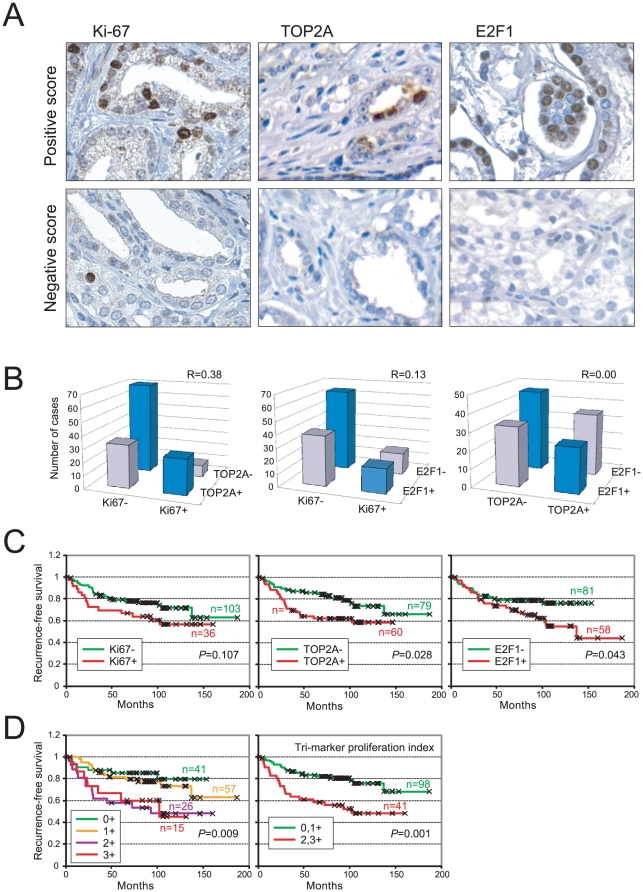
Immunostaining of Ki-67, TOP2A and E2F1 are prognostic. (**A**) Immunostaining of proliferation markers Ki-67, TOP2A and E2F1; representative positive and negative cases shown. (**B**) Pairwise comparison of immunostain scores across cases. Pearson correlation (R) values shown. (**C**) Kaplan-Meier analysis of Ki-67 (<5% *vs.* ≥5% tumor nuclei), TOP2A (<5% *vs.* ≥5% tumor nuclei) and E2F1 (<50% *vs.* ≥50% tumor nuclei) immunostaining. *P*-values (log-rank test) shown. (**D**) Kaplan-Meier analysis of combined marker staining (see keys). *P*-values (log-rank test) shown.

Immunohistochemistry was done on a tissue microarray that contained prostate cancer cases from radical prostatectomy (Table 1), associated with a median clinical follow-up of eight years. All three proliferation markers exhibited the expected nuclear staining (Fig. 2A). Interestingly, while Ki-67 and TOP2A typically stained only a small fraction of tumor nuclei, E2F1 often stained the majority of tumor cells (Fig. 2A). Ki-67 and TOP2A immunostaining across cases was significantly correlated (R = 0.38, *P*<0.001) (Fig. 2B). Ki-67 and E2F1 were less correlated (just missing statistical significance) (R = 0.13, *P* = 0.06), and somewhat surprisingly, TOP2A and E2F1 did not appear correlated (R = 0.00, *P* = 0.50) (Fig. 2B).

To evaluate and compare prognostic value, we performed Kaplan-Meier analyses on the set of 139 cases that were evaluable for all three proliferation markers. Ki-67 positivity (≥5% nuclei staining) showed a strong trend towards earlier biochemical-recurrence following prostatectomy (*P* = 0.107) (Fig. 2C) (analysis of the full set of 156 cases evaluable for Ki-67 reached statistical significance; data not shown). TOP2A positivity (≥5% nuclei staining) and E2F1 positivity (≥50% nuclei staining) were significantly associated with earlier recurrence (*P* = 0.028 and *P* = 0.043, respectively) (Fig. 2C). None of the three proliferation biomarkers was significantly associated with other clinicopathologic features, including preoperative PSA, Gleason grade and pathologic stage (Table 2).

**Table 2:** Association of proliferation markers with clinicopathologic variables.

Proliferation biomarker	Clinicopathologic parameter	P-value^a^
Ki-67	Preoperative PSA (≥10 *vs.* <10 ng/ml)	0.85
	Gleason grade (≥4+3 *vs.* ≤3+4)^b^	0.20
	Pathologic stage (≥T3a *vs.* ≤T2b)^c^	0.24
TOP2A	Preoperative PSA (≥10 *vs.* <10 ng/ml)	1.0
	Gleason grade (≥4+3 *vs.* ≤3+4)^b^	0.12
	Pathologic stage (≥T3a *vs.* ≤T2b)^c^	0.06
E2F1	Preoperative PSA (≥10 *vs.* <10 ng/ml)	0.73
	Gleason grade (≥4+3 *vs.* ≤3+4)^b^	0.82
	Pathologic stage (≥T3a *vs.* ≤T2b)^c^	0.39

a Two-sided Fisher's exact test.

b Stratification based on limited representation of Gleason 6 and 4+4 cases.

c Stratifies pathologic stage based on organ confinement.

We also determined whether combining markers might enhance prognostic value. A comparison of cases having no, one, two or three positive proliferation markers showed significant differences (*P* = 0.009), in general with incremental numbers of positive markers associated with earlier recurrence (Fig. 2D). Stratifying cases with no or one positive marker, *vs.* cases with two or three positive markers, hereafter termed the tri-marker “proliferation index”, provided the greatest prognostic significance (*P* = 0.001), with a hazard ratio of 2.6 (95% confidence interval 1.4–4.9). This result was also evident by analysis of ROC curves (Table 3; note the greater area under the curve for the tri-marker proliferation index).

**Table 3:** Receiver-operating characteristic (ROC) curve analysis.

Variable	Area under the curve^a^
Preoperative PSA (per ng/ml)^b^	0.658
Gleason grade (≥4+3 *vs.* ≤3+4)^c^	0.622
Pathologic stage (≥T3a *vs.* ≤T2b)^d^	0.690
Ki-67	0.572
TOPO2A	0.601
E2F1	0.599
Tri-marker proliferation index	0.642
Clinical model (PSA+grade+stage)	0.786
Clinical plus tri-marker proliferation index	0.816

a Evaluated at 8 years follow-up (the median follow-up time).

b Analyzed as a continuous variable.

c Stratification based on limited representation of Gleason 6 and 4+4 cases.

d Stratifies pathologic stage based on organ confinement.

**Figure 3:**
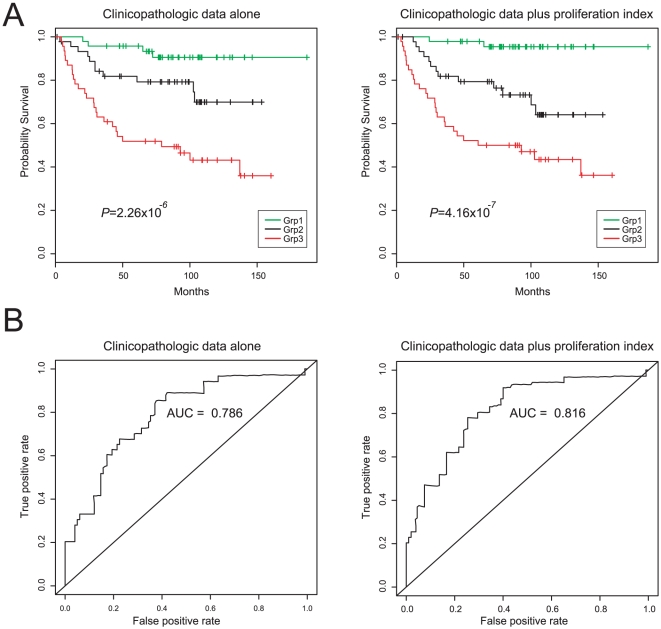
Incorporation of proliferation index improves prognostic value. (**A**) Kaplan-Meier analysis comparing multivariate models based on clinicopathologic data (*left*) and clinicopathologic data plus the tri-marker proliferation index (*right*). Cases are grouped by tertile. Log-rank test *P*-values are indicated. (**B**) ROC curve analysis comparing multivariate models based on clinicopathologic data (*left*) and clinicopathologic data plus the tri-marker proliferation index (*right*). Analysis done at 8 years follow-up (the median follow-up time for the cohort). Areas under the curve (AUC) are indicated.

Notably, in a multivariate analysis that included known prognostic markers – preoperative serum PSA, Gleason grade and pathologic stage, – the composite proliferation index remained a significant predictor (*P* = 0.005) (Table 4). The improved prognostication afforded by incorporating the proliferation index (compared to the clinicopathologic data alone) was confirmed by comparison of Kaplan-Meier plots (Fig. 3A; note the more significant log-rank test P-value) and of ROC curves (Table 3 and Fig. 3B; note the greater area under the curve).

**Table 4:** Analysis of PSA recurrence-free survival.

Univariate analysis	Hazard ratio (95% CI)	P-value^a^
Preoperative PSA (per ng/ml)^b^	1.04 (1.02–1.06)	<0.001
Gleason grade (≥4+3 *vs.* ≤3+4)^c^	3.37 (1.74–6.53)	<0.001
Pathologic stage (≥T3a *vs.* ≤T2b)^d^	4.56 (2.24–9.32)	<0.001
Tri-marker proliferation index	2.64 (1.43–4.87)	0.001

Multivariate analysis		
Preoperative PSA (per ng/ml)	1.04 (1.02–1.06)	<0.001
Gleason grade (≥4+3 *vs.* ≤3+4)	2.66 (1.36–5.20)	0.004
Pathologic stage (≥T3a *vs.* ≤T2b)	3.45 (1.68–7.10)	<0.001
Tri-marker proliferation index	2.47 (1.31–4.64)	0.005

a Log rank test (univariate analysis) or Wald test (multivariate analysis).

b Analyzed as a continuous variable.

c Stratification based on limited representation of Gleason 6 and 4+4 cases.

d Stratifies pathologic stage based on organ confinement.

## Discussion

In this study, we assessed for prostate cancer the prognostic utility of tissue biomarkers of cell proliferation, identified from expression profiling patterns. Two biomarkers, TOP2A and E2F1, scored by immunohistochemistry, were each prognostic (and the classic marker Ki-67 nearly so). Notably, combining markers further improved prognostic value. A composite proliferation index (0,1 *vs.* 2,3 positive markers) provided the greatest prognostic significance, and retained value when combined with currently used prognostic factors.

The markers we evaluated were selected from among the prostate cancer proliferation cluster genes. The “proliferation signature” (reviewed in ref. [23]) is a commonly encountered pattern in microarray studies. First reported in breast cancer cell lines and tumors [24], the proliferation signature is prominent across diverse cancer types (in comparison to normal tissue) [25]. Its membership significantly overlaps with cell-cycle regulated genes [26], and its presence correlates with cell doubling times in culture [27], and Ki-67 staining in tumors [24], [25]. The proliferation signature has also been found prognostic in breast cancer [28], [29] and in mantle cell lymphoma [30].

Gene signatures likely capture more information than assaying individual genes, and indeed multi-gene signature assays have reached clinical use [31]–[33]. Notably, two such breast cancer prognostic/predictive signatures include genes reporting on cell proliferation [31], [32]. However, microarray testing is not easily implemented in the clinical lab. We therefore asked whether a small number of genes might effectively capture the relevant information in the proliferation signature, by straight-forward immunohistochemistry.

We evaluated three proliferation genes. Ki-67 (proliferation-associated Ki-67 antigen), encoded by the *MKI67* gene and commonly identified by the MIB-1 antibody, is a nuclear antigen expressed in proliferating but not quiescent cells [34], though its specific function remains uncertain. Prior studies have shown Ki-67 to be an independent predictor of outcome in prostate cancer patients treated by radical prostatectomy [35]–[39] or radiotherapy [40]. Consistent with these studies, we also found Ki-67 to be prognostic, though it missed significance for the subset of cases evaluable for all three proliferation markers.

TOP2A is a type II DNA topoisomerase, controlling the topological state of DNA (by catalyzing transient double stranded breaks) during DNA transcription, recombination, replication, and chromosome partitioning at cell division [41]. TOP2A is also a target of several anti-neoplastic drugs, e.g. doxorubicin and etoposide. TOP2A expression by immunohistochemistry has been shown to be prognostic in breast [42] and ovarian [43] cancers. More recently, TOP2A transcript [44] and protein [45] levels have been associated with systemic progression of prostate cancer. Our own data corroborate this association.

E2F1 is a key transcriptional regulator of DNA replication and cell-cycle progression, and is negatively regulated by the RB1 tumor suppressor [46]. The Rb/E2F pathway is disrupted in most cancers [47]. Notably, a significant enrichment of E2F1 binding sites has been found among the promoter regions of proliferation signature genes [48], and known targets include for example *CCNE1*, *CDC25A*, *MCM4*, *TK1*, and *CENPE* [49]. Therefore, E2F1 is not only a proliferation signature gene, but itself a transcriptional regulator of other proliferation signature genes.

E2F1 immunostaining has been found to be prognostic in breast [50] and lung [51] cancers, and we now extend this to prostate cancer. Notably, while E2F1, Ki-67 and TOP2A transcript levels are all cell-cycle regulated [26], we generally observed E2F1 immunostaining in a larger proportion of tumor cells, in contrast to Ki-67 and TOP2A, which stained only a minority fraction of cells. As such, scoring E2F1 positivity might be more straightforward and reproducible, and further evaluation of E2F1 as a proliferation and prognostic marker in prostate and other cancer types is warranted.

Importantly, we found that scoring three proliferation markers provides superior prognostic information than scoring any single one. Why should this be so? At the transcript level, E2F1, TOP2A and Ki-67 peak at different stages of the cell cycle, G_1_/S, G_2_ and G_2_/M, respectively [26]. It is therefore possible that the three markers together provide a more complete snapshot of proliferating cells. Alternatively, using three markers might “smooth out” imprecision in pathologist scoring. Whether including more than three proliferation markers might further improve performance is unknown, though we note that three markers is within the range of what is practical for histology labs (where antibody panels are routinely ordered), and easier, quicker and cheaper than a microarray.

In summary, we have shown that a tri-marker proliferation index provides improved prognostic performance in prostate cancer, adding value above currently used prognostic factors. While validation in additional cohorts, and on whole tissue sections, is warranted, our findings suggest that the proliferation index might assist in risk stratification, for selecting appropriately aggressive therapies (e.g. use of adjuvant therapy) and ultimately improving patient outcomes. Cleary, clinical trials would be required to assess the benefit with regard to prostate cancer-associated morbidity and mortality. Finally, it is worth noting that many anti-neoplastic drugs target cell proliferation [23], including taxanes which show promise in the adjuvant/neoadjuvant setting [4]. Therefore, it is possible (and worth investigating further) that the proliferation index might also show utility in predicting response to specific chemotherapy.
